# Three Years Evaluation of Drug Shortages from Educational Pharmacies in Tehran

**Published:** 2012

**Authors:** Kheirollah Gholami, Golnaz Kamalinia, Mohammad Mahdi Ahmadian Attari, Jamshid Salamzadeh

**Affiliations:** a *Department of Clinical Pharmacy, Faculty of Pharmacy and Research Center for Rational Use of Drugs, Tehran University of Medical Sciences, Tehran, Iran.*; b *Department of Pharmaceutics, Faculty of Pharmacy, Tehran University of Medical Sciences, Tehran, Iran. *; c *Department of Traditional Pharmacy, Faculty of Traditional Medicine, Shahid Beheshti University of Medical Sciences, Tehran, Iran.*; d *Department of Clinical Pharmacy, School of Pharmacy, Shahid Beheshti University of Medical Sciences, Tehran, Iran.*

**Keywords:** Drug shortages, WHO essential list of drugs, Iran, National drug list, Ministry of health

## Abstract

The effectiveness of any drug supply systems in providing a trustworthy supply of essential drugs is a critical issue. To evaluate this effectiveness, it is necessary to watch over the status of the essential medicines in any country impartially and continuously. Some countries and also the World Health Organization (WHO) have codified a list of minimum medicines needed for a basic health care system and published them in assortments as a list of essential medicines. The aim of this study was to give an evaluation of the shortages status in Iran and identify the strengths and weaknesses of policies made in Ministry of Health during the years 2005 to 2008 in providing the essential drugs based on the WHO list of essential medicines.

The reports used in this retrospective study were collected from the central purchasing unit of one of the main chain drugstores in the country (13-Aban Pharmacy) every 2 to 3 weeks. In these reports, a drug is added to the list of shortages when the requested drug is not delivered. The reports were studied and the results were analyzed based on the WHO list of essential medicines and the national drug list of Iran. The shortages always included 20 to 40 medicines from the list of essential drugs compiled by WHO. Based on this finding, the Ministry of Health and particularly Food and Drug Organization can compile a National List of Essential Medicines and try to always supply them and prevent their shortage.

## Introduction

The effectiveness of drug supply systems in providing a reliable and trustworthy supply of essential medicines is a critical issue among different countries .The evaluation of the supply system of a country can be monitored via accessing the availability of essential medicines impartially and continuously. Drug shortages can frustrate the drug therapy process. Shortages can result in increased medical expenditures by increasing the probability of medical complications due to the lack of an appropriate therapy available at the proper time. Shortages can also result in more wasted time spent to attain drugs (1).

In Iran, we have faced with a significant incidence of drug shortages. These shortages have been reflected in 13-Aban pharmacy shortage reports. Tehran University of medical sciences, college of pharmacy, established seven community pharmacies in 1980s for its educational purposes that now are the major suppliers of medicine in Tehran, the capital of the country. These pharmacies have contracts with all main Iranian health insurance companies and they fill about thirty-five hundred prescriptions per day. Moreover, these pharmacies have an important role in the management of shortages since their establishment. In the case of shortages occurrence, distribution is usually centralized in these pharmacies to overcome insufficient supplies. Therefore, shortage reports of 13-Aban pharmacy are an alarm for the Ministry of Health (MOH) to be cautious about the shortages throughout the country.

With the above background, it is obvious that the shortage reports of 13-Aban pharmacy contain important information on the availability of medicines in the country. Analyzing these reports was the aim of this research project which can give clues about the status, strengths and weaknesses of Iran National Drug Policy (NDP) from March 2005 to 2008.

## Experimental

Shortage data from 2005 to 2008 were collected from the central purchasing unit of 13-Aban pharmacy. Drugs were listed in shortage reports after the order of pharmacy to purchase from wholesaler failed for at least three times. Drug shortages were identified by buying staff and were reported under the supervision of a pharmacist who was the manager of the central purchasing unit. The shortage reports did not follow a definite chronological pattern and they were reported only when the pharmacist realized that there are critical. In this study, these reports were assorted monthly and more evaluation was done by quantitative and qualitative count of their items. In quantitative count of drugs, numbers of drugs which were affected by the shortages during some days or all days of a month were counted. Additionally, the number of months in which a drug had faced the absolute shortage was counted. WHO has published a model list of essential medicines (2). To evaluate the importance of drug shortages of 13-Aban pharmacy, counted items were re-assorted on the basis of WHO essential medicines and the results of this assessment were compared with the results of original counts.

**Table 1:** Number of months that a medicine was affected by shortage from March 2005 to February 2008. Essential medicines based on WHO list are in bold

**Pharmacological categories**		**Drug names**	**No. months**
Anaesthetics		Lidocaine and Epinephrine, injection, 1%	14
		Lidocaine and Epinephrine, injection, 2%	6
Analgesics, Antipyretics, NSAIDs and DMARDs		Acetaminophen, tablet 80 mg	7
		Gold sodium thiomalate, injection, 25 mg/0.5 mL	5
		Gold sodium thiomalate, injection, 50 mg/0.5 mL	5
		Penicillamine, capsule, 250 mg	6
Antidotes and Other Substances Used In Poisonings		Methylene blue, injection, 100 mg/10 mL	6
		Penicillamine, capsule, 250 mg	6
Anticonvulsants/Antiepileptics		Diazepam suppository, 5mg	6
		Ethosuximide, syrup, 250 mg/5 mL	9
		Gabapentin, capsule, 100 mg	8
		Gabapentin, capsule, 300 mg	6
		Gabapentin, capsule, 400 mg	7
		Phenobarbital, injection, 200 mg/mL	5
Anti-Infective Medicines		Amphotericin B, injection, 50 mg	8
		Chloramphenicol, capsule, 250 mg	6
		Chloramphenicol, suspension 150 mg/ 5 mL	9
		Furazolidone, tablet, 100 mg	5
		Imipenem and Cilastatin, injection, 500 mg Imipenem, 500 mg Cilastatin	6
		Isoniazid, tablet, 300 mg	6
		Levamisole, syrup, 40 mg/ 5 mL	6
		Metronidazole, vaginal gel, 0.75%	10
		Nafcillin, injection, 1 g/vial	7
		Nitrofurantoin, tablet, 100 mg	7
		Nystatin, ointment, 10,000,000 units/100 g	4
		Paromomycin, capsule, 250 mg	4
		Paromomycin, syrup, 125 mg/5 mL	4
		Polymyxin B, Neomycin, Hydrocortisone, ear drops, 10000 U/ 1 mL Polymyxin B, 5 mg/1 mL Neomycin, 10 mg/1 mL Hydrocortisone	6
		Pyrimethamine, tablet, 25 mg	6
		Pyrvinium Pamoate, tablet, 50 mg	6
		Ribavirin, capsule, 200 mg	4
		Spiramycin, tablet, 500 mg	7
		Piperacillin and Tazobactam sodium, for injection, Piperacillin 2 g, Tazobactam 250 mg	7
		Piperacillin and Tazobactam sodium, for injection, Piperacillin 4 g Tazobactam 500 mg	9
		Tobramycin, injection, 40 mg/ mL (2 mL)	5
		Zidovudine, capsule,100 mg	10
Antineoplastic, Immunosuppressives and Medicines Used in Palliative Care		Melphalan, tablet, 2 mg		9
		Melphalan, injection, 50 mg		8
		BCG vaccine, injection, 50 mg		4
		Irinotecan, injection, 40 mg/2 mL		9
		Carmustine, injection, 100 mg/ vial		13
		Cyclosporine (Cicloral®), capsule, 100 mg		6
		Cyclophosphamide, tablet, 50 mg		6
		Oxaliplatin, injection, 50 mg		4
		Epirubicin, injection, 10 mg/ vial		5
		Epirubicin, injection, 50 mg/ vial		5
		Etoposide, injection, 100 mg/5 mL		6
		Chlorambucil, tablet, 2 mg		4
		Methotrexate, injection, 50 mg (non for intratechal administration)		9
		Procarbazine, capsule, 50 mg		5
		Teniposide, injection, 50 mg/ 5 mL		7
		Thioguanine, tablet, 40 mg		6
		Idarubicin, injection, 10 mg/ vial		7
Antiparkinsonism Medicines		Benserazide and Levodopa, tablet, 100 mg Levodopa, 25 mg Benserazide		7
		Benserazide and Levodopa, tablet, 200 mg Levodopa, 50 mg Benserazide		6
		Levodopa and Carbidopa, tablet, 100 mg Levodopa, 10 mg Carbidopa		8
		Levodopa and Carbidopa, tablet, 250 mg Levodopa, 25 mg Carbidopa		8
		Ropinirole, tablet 0.25 mg		6
		Pramipexole, tablet 0.25 mg		4
Antitussives and Expectorants		Clobutinol, oral drops, 60 mg/ mL		6
		Clobutinol, tablet, 40 mg		3
		Dextromethorphan, oral drops, 4 mg/ mL		4
		Dextromethorphan, tablet 15 mg		4
Blood Products and Plasma Substitutes		Albumin, injection, 20%		6
		Erythropoietin (Eprex®), injection, 2000 U		6
		Gamma globulin, injection, 2.5		8
		Gamma globulin, injection, 5		6
		Etherified starch (Hemaxel®), serum 2%		7
Cardiovascular Medicines		Flecainide, tablet, 100 mg		4
		Hydralazine, tablet, 10 mg		5
		Hydralazine, tablet 25 mg		8
		Isosorbide dinitrate, sublingual tablet, 5 mg		8
		Minoxidil, tablet, 10 mg		4
		Minoxidil, tablet, 5 mg		4
		Propafenone, tablet, 150 mg		11
		Propafenone, tablet, 300 mg		10
		Quinidine, tablet, 200 mg		8
		Phenoxybenzamine, capsule, 10 mg		4
		Terazosin, tablet, 2 mg		3
Dermatological Medicines (Topical)		Alpha ointment*		8
		Benzoyl peroxide, lotion 10%		10
		Benzoyl peroxide, lotion 5%		6
		Dexpanthenol cream 5%		4
		Fibrinolysin, (Elase®), ointment, 30 U/30 g Firinolysin, 20000 U/30 g Desoxyribonuclease		18
		Hydroquinone, cream, 2%		9
		Lindane, shampoo, 1%		4
		Mafenide, cream, 85 mg/g		5
		Mupirocin, topical ointment, 2%		4
Diagnostic Agents		Omnipaque, vial 300 mg I/ mL (50 mL)		5
		Omnipaque, vial 300 mg I/mL (10 mL)		5
		Omnipaque, vial 240 mg I/ mL (10 mL)		4
		Omnipaque, vial 240 mg I/mL (100 mL)		4
		Omnipaque, vial 300 mg I/ mL (20 mL)		4
Gastrointestinal Medicines		Hydrocortisone, (Colifoam®), retention enema, 100 mg/60 mL		5
		Belladonna, Phenobarbital, tablet, 19.4 mcg Atropine sulphate, 6.5 mcg Hyoscine HBr, 103.7 mcg Hyoscynamine sulphate, 16.2 mg Phenobarbital		6
		Dimethicone, oral drops, 40 mg/mL		7
		Hyoscine, suppository, 10 mg		5
		Thiethylperazin, injection 6.5 mg/mL		12
Hormones, Other Endocrine Medicines And Contraceptives		Progesterone (Cyclogest®), suppository 200 mg		5
		Follitropin Alfa (Gonal- F ®), injection, 150 U		3
		Chorionic Gonadotropin (Human), IR, for injection, 1500U		6
		Chorionic Gonadotropin (Human), IR, for injection, 500U		7
		Chorionic Gonadotropin (Human) (pregnyl®), for injection, 5000U		6
		Chorionic Gonadotropin (Human), IR, for injection, 5000U		5
		Human Menopausal Gonadotropin (Menogon®), for injection, 75U FSH, 75U LH		3
		Human Menopausal Gonadotropin (Menopur®), for injection, 75U FSH, 75U LH		6
		Medroxyprogesterone, injection 150 mg/mL		7
		Estrogens (Conjugated/ Equine) (Premarin ®), injection, 25 mg/5 mL		7
		Gonadorelin (Stimu- LH®), for injection, 0.1 mg		6
		Protirelin (Stimu- TSH®), injection, 0.2 mg/mL		7
Immunologicals		Hepatitis B immune globulin, injection, 5% (1mL)		3
		Interferon Beta-1b (Betaferon®), injection, 0.3 mg		4
		Interferon Beta-1a (Rebif®), injection, 44 mcg/0.5 mL		3
Infant Formula		Milk Galantamin 17		8
		Milk Multi AR		7
		Milk Multi HA		7
		Milk SMA Wysoy		8
Medicines For Vascular Disorders		Citicoline, injection, 250 mg/2 mL		4
		Calcium dobesilate (Doxium®), tablet 250 mg		7
		Sodium Polystyrene Sulfonate, powder for suspension, 454g		5
Psychotherapeutic Medicines		Venlafaxine, tablet, 150 mg		5
		Venlafaxine, tablet, 37.5 mg		5
		Venlafaxine, tablet, 75 mg		5
		Haloperidol, oral drops, 2 mg/mL		7
		Promethazine tablet, 25 mg		9
		Tetrabenazine, tablet, 25 mg		5
		Tranylcypromine, tablet, 10 mg		8
Solutions Correcting Water, Electrolyte and Acid-Base Disturbances		Potassium chloride, tab 500 mg		9
		Magnesium sulphate, vial10%		12
		Magnesium sulphate, vial20%		6
		Magnesium sulphate, vial50%		5
		Sodium Bicarbonate, for infusion, 7.5%		5
Vitamins, Minerals, Dietary Supplements, And Caloric Agents		Dihydrotachysterol, oral drops, 1 mg/mL		5
		Calcium folinate, amp 3 mg/mL		10
		Fresubin® (chocolate)		6
		Fresubin® (straw berry)		6
		Magnesium gluconate, tab 500 mg		4
		Magnesium gluconate, tab 0.22 mg		4
		Sodium fluoride, tab 20 mg		5
		Sodium fluoride, tab 1 mg		3
		Monobasic Sodium Phosphate (Phosphate Sandoz®), effervescent tablet		7
Miselanous		Neostigmine, injection, 2.5 mg/mL		9
		Sodium benzoate, injection, 2 mg		10
		Sodium benzoate, tablet 500 mg		4
		Tizanidine, tablet, 4 mg		4

* Alpha ointment is a burning ointment which has a natural source formula (*Lawsonia inermis*) and promotes healing and reduces scarring

## Results and Discussion

In this project, the shortage reports of the central purchasing unit in 13-Aban pharmacy dated between 2005 and 2008 were analyzed. The quantitative and qualitative overview of the data was done by the use of Iranian National Drug List (NDL) and WHO model list of essential medicines respectively (3).

As it is presented in Table 1, the qualitative count of drug shortages shows 146 items affected with shortages during the years 2005-2008. Some of these items like fibrinolysin (ointment), lidocaine (1% vial) and carmustine (100 mg vial) had been cumulatively unavailable for more than one year. Comparing the items of this table with WHO list of essential medicines shows that 35 of 146 drug items were among essential pharmaceuticals. During these three years, anti-infective and antineoplastic medicines with 22 and 18 items respectively, had the highest shortages among the pharmaceuticals. Some broad spectrum antibiotics like Imipenem/cilastatin (500 mg vial) and piperacillin/tazobactam (2.25 and 4.5 mg vials) were among these medicines.

Because of the retrospective nature of this investigation, we encountered the following problems; I: All shortage reports of 13-Aban pharmacy are kept in paper sheet files and therefore, transforming data to Excel format was time consuming and tedious. II: Another problem was the missing of data sheets in archives. III: The shortages were reported when the manager realized that they are critical and therefore, it did not follow a definitive chronological.

Furthermore, 13-Aban is a community pharmacy and does not dispense all pharmaceuticals. Some of the pharmaceuticals like vaccines for routine immunization, narcotics and antivenins are not distributed in this pharmacy. Therefore, following the shortages of these items was not possible with this project.

In the anti-parkinsonism medicines group, it can be observed that the four major drugs which are used in the treatment of these patients in Iran were affected by shortages for at least 6 months during these years, which have resulted in several switches between these drugs and therefore, a potential decrease in the patients’ compliance may have occurred. Two widely used beta interferons, Betaferon® and Rebif®, which are prescribed for the multiple sclerosis patients have shown shortages for 4 and 3 months respectively. Any shortages in this group of pharmaceuticals is especially important since the shortages can exert the stress on patients and it has been shown that Multiple sclerosis exacerbations may become more common after the stressful life events and a significant association between stress and MS relapses has been observed (4-6).

**Figure 1:**
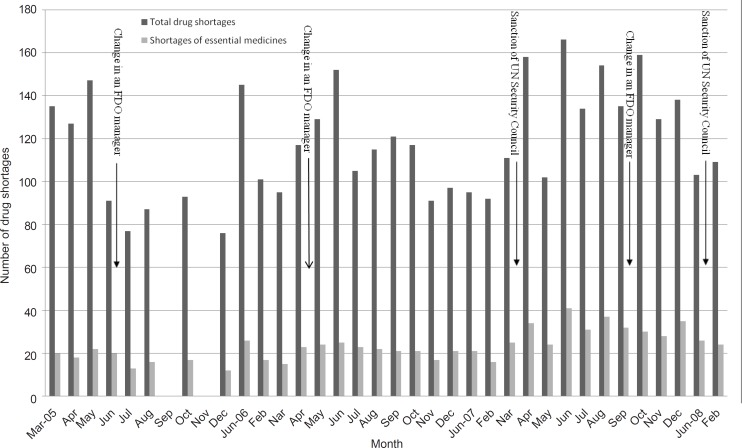
Quantitative evaluation of drug shortages of 13-Aban pharmacy from March 2005 to February 2008. Number of drug shortages is presented as the total drug shortages (dark gray columns) and shortages that affected the essential medicines (light gray columns).

There has not been a standard or a check list to determine the frequency of drug shortages. Therefore, shortages have different meanings for people with different perspectives. Most health care organizations consider the problems of the supply chain system as a major cause of drug shortages. These shortages can influence the patient care definitely, especially when the prescriber have to choose an alternative therapy (7). In this study, our focus was on supply issues. Databases used to compile the shortage reports were the back orders of the purchasing requests made by 13-Aban central purchasing unit. In accordance with these reports, quantitative and qualitative counts of shortages were done and the results of them are presented in Figure 1 and Table 1.

A drug shortage usually does not have a single cause. It can be due to the manufacturing, marketing, economical, distribution and regulatory problems (8). The source of data in this project was limited to the central purchasing unit’s back orders of 13-Aban pharmacy and therefore, the results could make conclusions on the signs and not the exact causes of shortages. Compiling other information has helped addressing the possible causes of these shortages. Comparing the chart of shortages with circumstantial events can be useful in finding some of the reasons for shortages. As it is shown in the Figure 1, there are relationships among some of the chart’s peaks with circumstantial events like United Nations (UN) Security Council bans and replacing the drug regulators. It seems possible that the regulation and enforcement problems be one of the reasons of the fluctuations in the drug market.

Many countries and also the WHO have codified a list of minimum medicines needed for a basic health care system and published them in assortments as a list of essential medicines. Essential medicines are intended to be available within the context of functioning health care systems at all times in adequate amounts, in the appropriate dosage forms, with assured quality, and at a price which the individual and the community can afford (2). Iran has got NDL instead of essential drugs’ list. Publishing NDL makes Food and Drug Organization (FDO) responsible for preparing all of the items in the list. In this project, analyzing the shortages was done according to both NDL and WHO model list of essential medicines. When the evaluation of the shortage is based on the WHO list of essential medicines, the shortages are not as substantial as the time when the evaluation is based on NDL. Although the shortage reports on the basis of WHO essential medicines exhibited the considerable decrease in the number of shortages, it also showed continuous shortage of some essential medicines. Compiling Iranian list of essential medicines can make FDO capable of monitoring and managing shortages more easily and accurately and let FDO to focus on the shortages which are critical for the health care system.

To summarize, as it was shown in this study, Iran faced considerable drug shortages between the years 2005-2008. It should be reminded that the research was carried out in a limited period of time and it does not consider the shortages before and after this period; although drug shortages out of this period of time may be considerable either. Our qualitative survey, however, showed that many of such shortages were not with regard to WHO essential medicines. The probable causes of Iranian drug shortages are possibly regulation and enforcement problems, continuous changes in FDO managers, and UN Security Council bans. It seems that establishing consistency in FDO directorship, improving some of Iran drug policies and compiling the list of Iranian essential medicines will be effective in controlling drug shortages.
